# Prevalence of inherited neurotransmitter disorders in patients with movement disorders and epilepsy: a retrospective cohort study

**DOI:** 10.1186/s13023-015-0234-9

**Published:** 2015-02-08

**Authors:** Saadet Mercimek-Mahmutoglu, Sarah Sidky, Keith Hyland, Jaina Patel, Elizabeth J Donner, William Logan, Roberto Mendoza-Londono, Mahendranath Moharir, Julian Raiman, Andreas Schulze, Komudi Siriwardena, Grace Yoon, Lianna Kyriakopoulou

**Affiliations:** Division of Clinical and Metabolic Genetics, Department of Pediatrics, University of Toronto, The Hospital for Sick Children, Toronto, Canada; Genetics and Genome Biology, Research Institute, The Hospital for Sick Children, Toronto, Canada; Medical Neurogenetics, LLC, Atlanta, GA USA; Division of Neurology, Department of Pediatrics, University of Toronto, The Hospital for Sick Children, Toronto, Canada; Biochemical Genetics Laboratory, Department of Laboratory Medicine, University of Toronto, The Hospital for Sick Children, Toronto, Canada; Division of Clinical and Metabolic Genetics, Department of Pediatrics, University of Toronto, Genetic and Genome Biology, Research Institute, The Hospital for Sick Children, 555 University Avenue, Toronto, ON M5G 1X8 Canada

**Keywords:** Inherited neurotransmitter disorders, Genetics, Movement disorders, Monoamine metabolism, Pyridoxine metabolism, Epilepsy

## Abstract

**Background:**

Inherited neurotransmitter disorders are primary defects of neurotransmitter metabolism. The main purpose of this retrospective cohort study was to identify prevalence of inherited neurotransmitter disorders.

**Methods:**

This retrospective cohort study does not have inclusion criteria; rather included all patients who underwent cerebrospinal fluid (CSF) homovanillic and 5-hydroxyindol acetic acid measurements. Patients with CSF neurotransmitter investigations suggestive of an inherited neurotransmitter disorder and patients with normal or non-diagnostic CSF neurotransmitter investigations underwent direct sequencing of single gene disorders.

**Results:**

There were 154 patients between October 2004 and July 2013. Four patients were excluded due to their diagnosis prior to this study dates. Two major clinical feature categories of patients who underwent lumbar puncture were movement disorders or epilepsy in our institution. Twenty out of the 150 patients (13.3%) were diagnosed with a genetic disorder including inherited neurotransmitter disorders (6 patients) (dihydropteridine reductase, 6-pyruvoyl-tetrahydropterin synthase, guanosine triphosphate cyclohydrolase I, tyrosine hydroxylase, pyridoxine dependent epilepsy due to mutations in the ALDH7A1 gene and pyridoxamine-5-phosphate oxidase deficiencies) and non-neurotransmitter disorders (14 patients).

**Conclusion:**

Prevalence of inherited neurotransmitter disorders was 4% in our retrospective cohort study. Eight out of the 150 patients (5.3%) had one of the treatable inherited metabolic disorders with favorable short-term neurodevelopmental outcomes, highlighting the importance of an early and specific diagnosis. Whole exome or genome sequencing might shed light to unravel underlying genetic defects of new inherited neurotransmitter disorders in near future.

## Background

**Figure 1:**
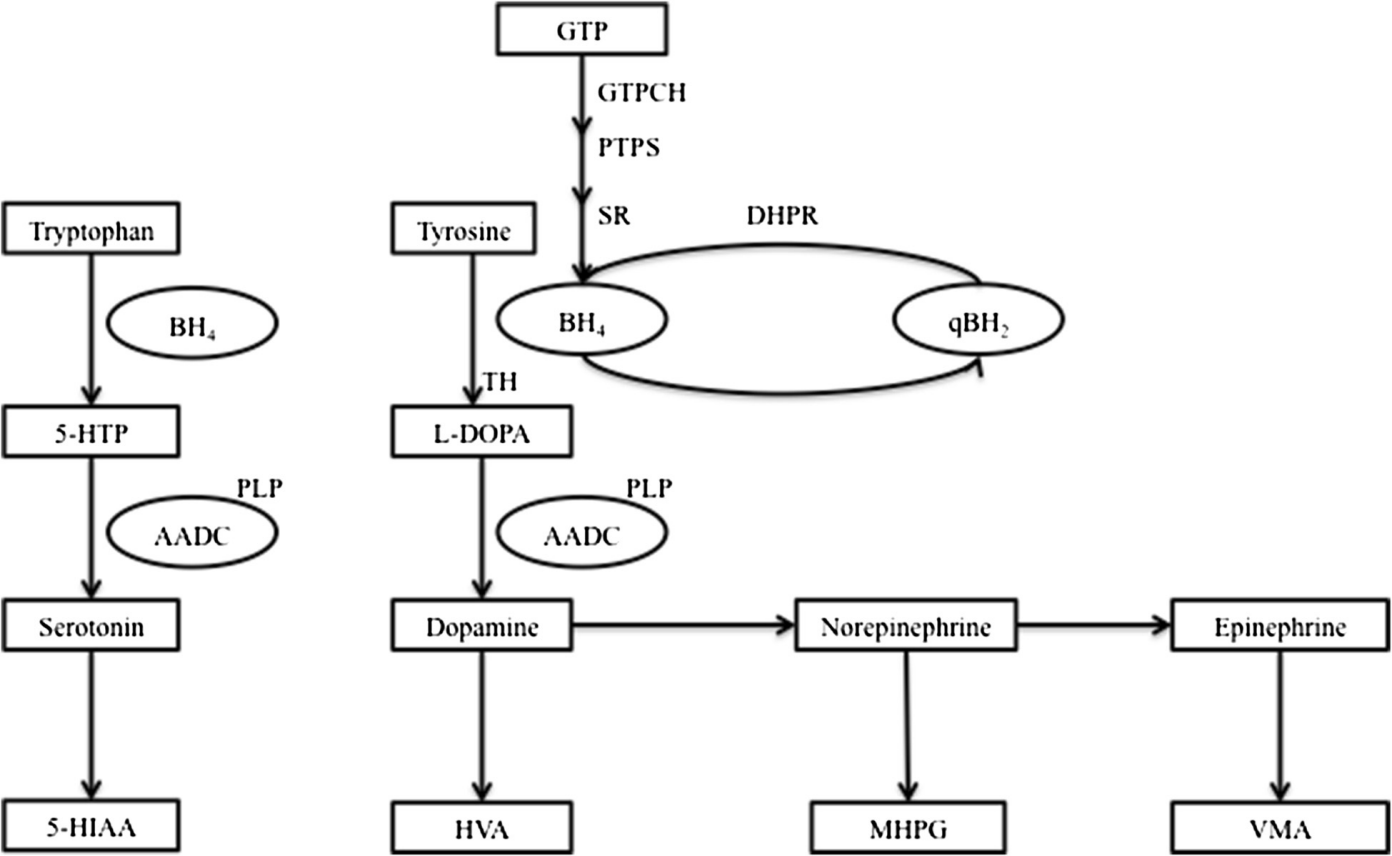
**The monoamine neurotransmitter pathway.** Abbreviations: GTP = guanosine triphosphate; GTPCH = guanosine triphosphate cyclohydrolase 1; PTPS = 6-pyruvoyltetrahydropterin synthase; SR = sepiapterin reductase; BH4 = tetrahydrobiopterin; TH = tyrosine hydroxylase; DHPR = dihydropteridine reductase; qBH_2_ = quinonoid dihydrobiopterin; 5-HTP = 5-hydroxytryptophan; L-dopa = levodihydroxyphenylalanine; PLP = pyridoxal phosphate; AADC = aromatic L-amino acid decarboxylase; 5-HIAA = 5-hydroxyindoleacetic acid; HVA = homovanillic acid; MHPG = 3-methoxy-4-hydroxylphenylglycol; VMA = vanillylmandelic acid.

Inherited neurotransmitter disorders are primary defects of neurotransmitter metabolism and transport. They include defects of catecholamine, serotonin, biopterin, glycine, pyridoxine and gamma amino butyric acid (GABA) metabolism [1,2]. Defects of catecholamine (dopamine, epinephrine and norepinephrine) and serotonin metabolism, also called monoamine or biogenic amine metabolism, are the most widely known and investigated group of neurotransmitter disorders [3-7]. Two cofactors are required for the synthesis of dopamine and serotonin: tetrahydrobiopterin and pyridoxal 5′-phosphate [4,5]. The end products of dopamine, serotonin, and norepinephrine within the central nervous system (CNS) are homovanillic acid (HVA), 5-hydroxyindolacetic acid (5-HIAA) and 3-methoxy 4-hydroxypenylglycol (MHPG), respectively (Figure 1). The cerebrospinal fluid (CSF) diagnostic metabolites used for diagnosis of defects of tetrahydrobiopterin metabolism are tetrahydrobiopterin, biopterin, and neopterin [8].

Tyrosine hydroxylase (TH) deficiency affects the synthesis of dopamine, epinephrine and norepinephrine, whereas aromatic L-amino acid decarboxylase (AADC) and tetrahydrobiopterin metabolism defects including guanosine triphosphate cyclohydrolase I (GTPCH), dihydropteridine reductase (DHPR), 6-pyruvoyltetrahydropterin synthase (PTPS), sepiapterin reductase (SR) deficiencies lead to a deficiency of serotonin in addition to deficiencies of dopamine, epinephrine and norepinephrine [1-5].

Lumbar punctures are widely used among patients with neurological signs and symptoms such as seizures, developmental delay, central hypotonia, and movement disorders including chorea, choreoathetosis, dystonia, dyskinesia, and ataxia [9-14]. The GTPCH, PTPS, DHPR and SR deficiencies are treatable. In patients with severe phenotype of AADC and TH deficiencies, treatments alleviate symptoms but do not affect neurodevelopmental disabilities. As the majority of inherited neurotransmitter disorders are treatable, CSF neurotransmitter measurements would be necessary to diagnose these disorders for the initiation of the disease specific treatment.

There are no incidence studies for inherited neurotransmitter disorders in the general population. It is also unknown if these disorders are under-diagnosed due to the nonspecific constellation of symptoms, as well as the cumbersome collection of CSF samples and lack of availability of metabolite measurements in laboratories in North America and Europe.

We performed a retrospective cohort study to investigate the diagnostic yield and the prevalence of inherited neurotransmitter disorders in patients who underwent lumbar puncture for the measurement of CSF HVA and 5-HIAA at The Hospital for Sick Children.

## Methods

This retrospective cohort study was approved by Institutional Research Ethics Board (REB #1000032023). All patients originating from The Hospital for Sick Children, Toronto, Canada were included who had the measurement of CSF HVA and 5-HIAA in the Medical Neurogenetic Laboratory in Atlanta. This laboratory is the reference clinical laboratory for the CSF neurotransmitter measurements for our hospital. Their database was searched using The Hospital for Sick Children to identify patient names for this retrospective cohort study. As we have no database or registry of patients who underwent lumbar puncture for CSF neurotransmitter measurements in our institution, this was the only way to develop patient cohort for this retrospective study. For these reasons, this study does not have inclusion criteria; rather included all patients originating from The Hospital for Sick Children who underwent CSF neurotransmitter measurements in a single laboratory for the diagnostic work-up. The main purpose of this retrospective cohort study was to identify prevalence of inherited neurotransmitter disorders.

The demographic, clinical, biochemical, molecular genetics and neuroimaging features were reviewed from electronic patient charts for all patients. Briefly, CSF samples were collected into pre-prepared tubes numbered from 1 to 5 according to reference clinical laboratory’s sample collection requirements. Dithioerythritol and diethylenetriaminepenta-acetic acid were added to the tube #3 to prevent the oxidation of tetrahydrobiopterin [15]. Samples were placed on ice and transferred immediately into the laboratory for freezing at −80°C until measurements. CSF samples contaminated with blood due to traumatic sample collection were centrifuged in cold centrifuge immediately and clear CSF sample was transferred into new tubes as described previously [3]. All samples were shipped to Medical Neurogenetics Laboratory in Atlanta on dry ice for the CSF neurotransmitter measurements. HVA, 5-HIAA and 3-OMD levels were measured in the first 0.5 mL of CSF sample. If HVA and 5-HIAA levels were abnormal, tetrahydrobiopterin and neopterin levels were measured in the third sample. Measurements were obtained using high-performance liquid chromatography with a combination of electrochemical and fluorescence detectors [3]. Metabolite levels were compared against age-appropriate reference ranges. CSF glucose and CSF 5- methylenetetrahydrofolate levels were also measured in some of the patients.

Patients with positive newborn screening for phenylketonuria (PKU) underwent sapropterin loading test, urine pterin metabolites and measurement of DHPR enzyme activity on dried blood spots in the neonatal period. Patients with normal or non-diagnostic CSF neurotransmitter metabolite measurements underwent direct sequencing of genes based on clinical (including paroxysmal events, hereditary spastic paraplegia, epilepsy, severe hypotonia or positive family history) or biochemical features (including elevated homocysteine, low CSF glucose, low CSF 5-methylenetetrahydrofolate levels) suggestive of single gene disorders or targeted next-generation sequencing for epileptic encephalopathy gene panel in various clinical molecular genetics laboratories.

All information was entered into an Excel database for analysis. The Alamut database, Exome database Variant Server (http://evs.gs.washington.edu/EVS/), and dbSNP database (http://www.ncbi.nlm.nih.gov/projects/SNP/) were used for the annotation of novel genetic variants. Pathogenicity of missense variants were assessed by cross-species conservation of nucleotides and amino acid sequences using gene and protein sequences from the Alamut database. *In silico* splice tools were used to test the potential effects of new synonymous or intronic variations on premRNA splicing. We used the recommendations for mutation nomenclature (www.hgvs.org/mutnomen) to name gene variations.

## Results

**Figure 2:**
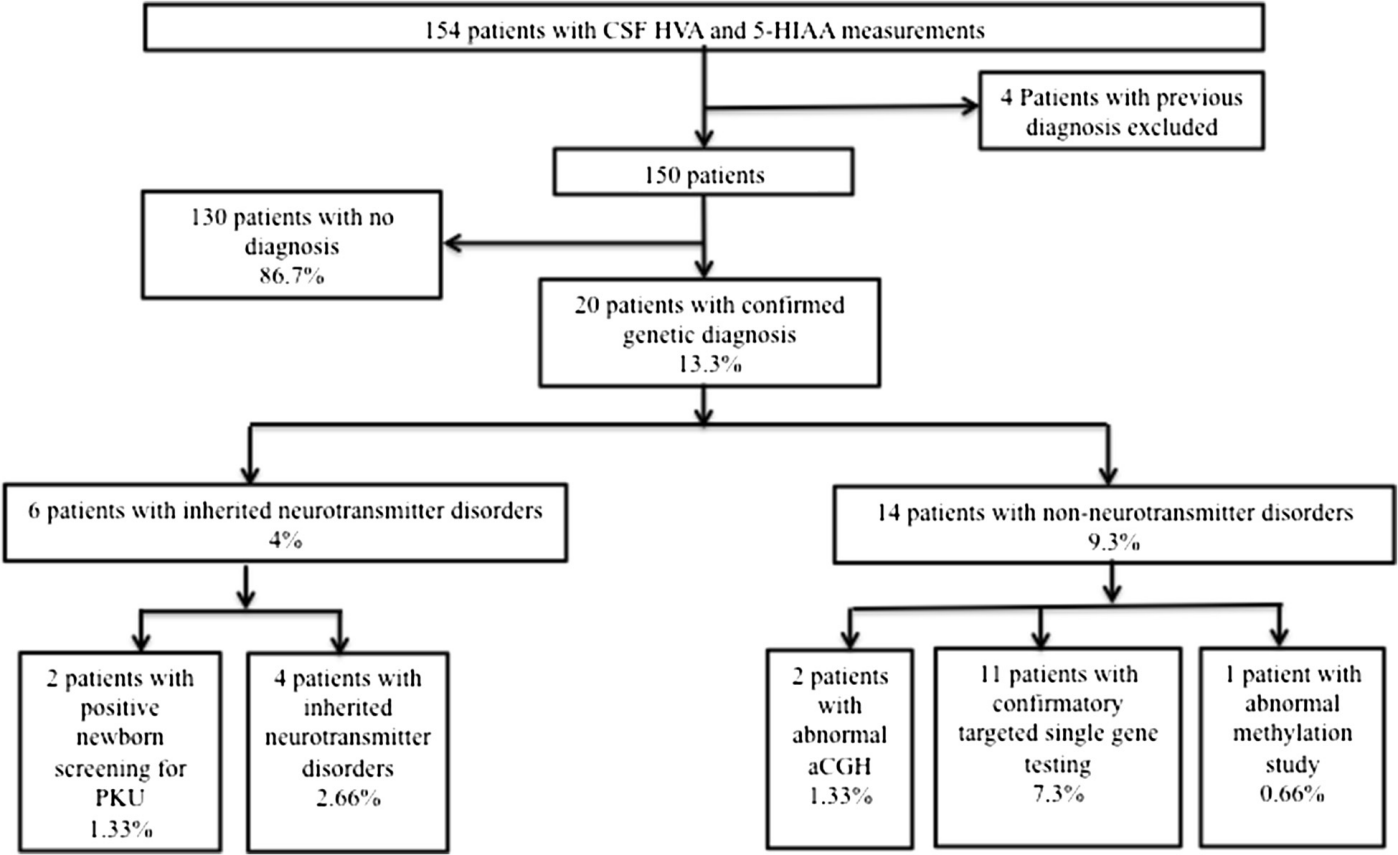
**Distribution of 154 study patients with or without genetic diagnosis.**

154 patients underwent lumbar puncture for CSF HVA and 5-HIAA at The Hospital for Sick Children between October 2004 and July 2013. Four patients were excluded because they had diagnosis of DHPR deficiency and underwent CSF HVA and 5-HIAA measurements for the guidance of the treatment during this retrospective cohort study period. The remaining 150 patients were included in this study. Two major clinical feature categories of patients who underwent lumbar puncture were movement disorders or epilepsy in our institution. There was no diagnosis in 130 patients and there was a confirmed underlying genetic diagnosis in 20 patients (Figure 2). Patients with a confirmed genetic diagnosis were divided into 2 groups: 1) Inherited neurotransmitter disorders including catecholamine, serotonin, tetrahydrobiopterin and pyridoxine metabolism disorders (6 patients, 4%); 2) Non-neurotransmitter disorders with a confirmed underlying genetic diagnosis (14 patients, 9.3%).

The demographics, clinical features and their distribution and the number of the patients with a confirmed diagnosis for neurotransmitter and non-neurotransmitter disorders are listed in Tables 1 and 2 respectively.

**Table 1:** **Demographics and clinical features of the patients with inherited neurotransmitter disorders and non-neurotransmitter disorders**

**Demographics of the patients**	**Inherited neurotransmitter disorders**	**Non-neurotransmitter disorders**
**Number of patients**	6	14
**Average age of onset (range)**	3.8 ± 6.0 SD months (1 day to 17 months)	10.5 ± 16.2 SD months (1 day to 60 months)
**Average age of diagnosis (range)**	26.5 ± 42.6 SD months (1 to 120 months)	75.3 ± 64.8 SD months (7 to 216 months)
**Current average age (range)**	5.8 ± 4.4 SD years (1 to 14 years)	10.3 ± 6.4 SD years (3 to 19 years)
**Inheritance pattern**	AR (5 patients, 83%) AD (1 patient, 17%)	AR (6 patients, 43%) AD (5 patients, 36%) X-linked (1 patient, 7%) Other (2 patients, 14%)
**Clinical features**		
**Newborn screening positive for PKU**	2 patients (33%)	None
**Movement disorder and/or GDD**	2 patients (33%)	5 patients (36%)
**GDD and/or epilepsy**	2 patients (33%)	8 patients (57%)
**Neonatal hypotonia**	None (0%)	1 patient (7%)

*Abbreviations*: *SD* standard deviation, *AR* autosomal recessive, *AD* autosomal dominant, *GDD* global developmental delay.

**Table 2:** **Clinical features and diagnostic yield of the patients who underwent CSF neurotransmitter measurements including the inherited neurotransmitter and non-neurotransmitter disorders**

**Category of clinical features**	**Patient number (percent)**	**Patient number for inherited neurotransmitter disorders**	**Patient number for non-neurotransmitter disorders**
Movement disorder and/or GDD	38 (25%)	2 patients	1 patient
GDD and movement disorder and epilepsy	18 (12%)	None	4 patients
GDD and epilepsy	66 (44%)	2 patients	7 patients
GDD and hypotonia	26 (17.5%)	None	2 patients
Newborn screening positive for PKU	2 (1.5%)	2 patients	None

*Abbreviations*: *GDD* global developmental delay, *PKU* phenylketonuria.

### Inherited neurotransmitter disorders

There were 6 patients from 6 unrelated families in this group. Their clinical, biochemical, neuroimaging, molecular genetic investigations, and treatment outcome are summarized in Table 3.

**Table 3:** **Patients with confirmed inherited neurotransmitter disorders**

**Patients/sex/current age/diagnosis**	**Clinical features/age of onset/age of diagnosis**	CSF* HVA/5-HIAA/ 3-OMD/THB/neo/other (LP age)	**Molecular genetic testing** ^**reference**^	**Treatment/outcome**
1/M/9 yr/DHPR def	NBS-Pos-PKU/7 d/<15 d	**↓123**/**↓55**/**↑2441/ ↓14** (3.5 yr) on treat	Homozygous novel IVS5+ 1 G > T in *QDPR* gene	L-dopa/carbidopa 9 mg/kg/d,5-HTP 7.5 mg/kg/d/severe GDD
2/F/3 yr/PTPS def	NBS-Pos-PKU/6 d/12 d	**↓157**/**↓78**/NP/**↓ < 5**/ **↑123** N (12 d)	Homozygous known [16] c.200C > T (p.Thr67Met) in *PTS* gene	L-dopa/carbidopa 7.13 mg/kg/d,5-HTP 2.59 mg/kg/d, sapropterin 2 mg/kg/d)/normal
3/F/14 yr/GTPCH def	GM delay, ataxia, tremor/17 mo/10 yr	**↓121**/N/N/**↓8**/**↓ < 5** (13 yr)	Heterozygous known [17] c.225_225insA (pY75X) in *GCH1* gene	L-dopa/carbidopa 8.5 mg/kg/d/executive dysfunction, intermittent dystonia, seizures
4/F/3 yr/TH def	GDD, choreoathetosis, dystonia/2 mo/24 mo	**↓7**/N/N (2 yr)	Homozygous known [18] c.943G > A (p.Gly315Ser) in *TH* gene	L-dopa/carbidopa 7.5 mg/kg/d/severe GDD, spasticity
5/F/5 yr/PDE-*ALDH7A1*	Seizures, GDD/3.5 mo/11 mo	**↓175**/**↓110**/N (7 mo)	Homozygous novel IVS-12(+1) G > A in *ALDH7A1* gene	Pyridoxine 200 m/d/seizure free, severe GDD
6/F/1 yr/PNPO def	Neonatal MS, GTS/1 d/2 mo	N/N/**↑329/↑threonine 107** (3 d)	Homozygous known [19] c.448_451del (p.Pro150Argfs*27) in *PNPO* gene	PLP 35 mg/k/d/seizure free, normal development

*Abbreviations*: *CSF* cerebral spinal fluids, *HVA* homovanillic acid, *5-HIAA* 5-hydroxyindol acetic acid, *3-OMD* 3-O-methyldopa, *LP* lumbar puncture, *THB* tetrahydrobiopterin, *Neo* neopterin, *DHPR* dihydropterine reductase, *def* deficiency, *GA-I* glutaric aciduria type I, *NBS-Pos-PKU* newborn screening positive for phenylketonuria, *d* day(s), *mo* month(s), *GDD* global developmental delay, *NP* not performed, *ProReD* protein restricted diet, *5-HTP* 5-hydroxytryptophane, *PKU* phenylketonuria, *PTPS* 6-pyruvoyl-tetrahydropterin synthase, *GTPCH* guanosine triphosphate cyclohydrolase, *GM* gross motor, *TH* tyrosine hydroxylase, *PDE-* *ALDH7A1* pyridoxine dependent epilepsy caused by mutations in the *ALDH7A1* gene, *PNPO* pyridox(am)ine-5-phosphate oxidase, *MS* myoclonic seizures, *GTS* generalized tonic seizures, *PLP* pyridoxal-5-phosphate, *yr* year(s), *GTCS* generalized tonic-clonic seizures. 3: ; ↑ = elevated; ↓ = decreased.

*Age appropriate reference ranges for CSF neurotransmitters and amino acids: *CSF HVA:* 0–0.2 years = 337–1299 nmol/L; 0.2 - 0.5 years = 450–1132 nmol/L; 0.5 – 2 years = 294 – 1115 nmol/L; 2 – 5 years = 233–928 nmol/L; 5 – 10 years = 218–852 nmol/L; 10 – 15 years = 167–563 nmol/L; adults = 145–324 nmol/L.

*CSF 5HIAA*: 0–0.2 years = 208 – 1159 nmol/L; 0.2 - 0.5 years = 179 – 711 nmol/L; 0.5 - 2 years = 129 – 520 nmol/L; 2 – 5 years = 74 – 345 nmol/L; 5 – 10 years = 66 – 338 nmol/L; 10 – 15 years = 67 – 189; adults = 67–140 nmol/L.

*CSF 3-O-MD*: 0–0.2 years = <300 nmol/L; 0.2 - 0.5 years = <300 nmol/L; 0.5 – 2 years = <300 nmol/L; 2 – 5 years = <150 nmol/L; 5 – 10 years = <100 nmol/L; 10 – 15 years = <100 nmol/L; adults = <100 nmol/L.

*CSF tetrahydrobiopterin*: 0–0.2 years = 40–105 nmol/L; 0.2-0.5 years = 23–98 nmol/L; 0.5-2 years = 18–58 nmol/L; 5–10 years = 9–40 nmol/L; 10–15 years = 9–32 nmol/L; adults = 10–30 nmol/L.

*CSF neopterin*: 0–0.2 years = 7–65 nmol/L; 0.2-0.5 years = 7–65 nmol/L; 0.5-2 years = 7–65 nmol/L; 2–5 years = 7–65 nmol/L; 5–10 years = 7–40 nmol/L; 10–15 years = 8–33 nmol/L; adults = 8–28 nmol/L.

*CSF threonine*: 28–92 μmol/L.

In this group, 2 patients (33%) were identified by positive newborn screening for PKU: one had PTPS deficiency and another one had DHPR deficiency in the neonatal period based on the response to sapropterin loading test, or non-detectable DHPR enzyme activity on dried blood spots or elevated urine neopterin and neopterin to biopterin ratio. In 3 out of 6 patients (50%), the diagnosis was suspected based on the abnormal CSF HVA and/or 5-HIAA levels leading to diagnosis of the following inherited neurotransmitter disorders including autosomal dominant GTPCH deficiency, TH deficiency, pyridoxine dependent epilepsy (PDE) caused by mutations in the *ALDH7A1* gene (PDE-*ALDH7A1)* (Table 2). In one patient with PNPO deficiency (10%), mildly elevated CSF threonine and 3-OMD levels as well as response to pyridoxal-5-phosphate therapy led the confirmation of the diagnosis [19]. Two patients (33%) presented with a movement disorder (one patient with ataxia and tremor; one patient with dystonia and choreoathetosis), and two patients (33%) presented with GDD and epilepsy.

We identified 6 variants (2 novel and 4 known) in 6 genes including *QDPR, PTS, GCH1, TH, ALDH7A1* and *PNPO* genes in 6 patients*.* Five patients (83%) were homozygous for the variants (Table 3). In all patients with autosomal recessive disorder, carrier status was confirmed in both parents. None of the novel variants were found in either ESP6500 Exome or dbSNP database as polymorphisms. The variants were highly conserved across all species and reported to be deleterious in SIFT and/or Mutation Taster prediction programs, and all were likely disease causing mutations. Missense mutations, small deletion/insertions and splice site variants were equally occurred (Table 3).

In this group, 5 patients (83%) had a treatable inherited neurotransmitter disorders including GTPCH deficiency, PTPS deficiency, DHPR deficiency, PDE-*ALDH7A1* and PNPO deficiency. Despite strict treatment recommendations from the newborn period, the patient with DHPR deficiency had severe global developmental delay and cognitive dysfunction at the age of 9 years due to non-adherence to diet and medication treatment by parents. The treatment improved choreoathetoic movement disorder in the very severe TH deficiency patient, but did not change the severity of neurocognitive dysfunction and spasticity.

Additionally, 4 patients with a genetically confirmed diagnosis of DHPR deficiency who were diagnosed prior to October 2004 were reviewed for long-term treatment outcome, but not included into the prevalence calculations. A 15-year-old female with therapeutic phenylalanine levels by protein restricted diet and excellent compliance to l-dopa/carbidopa, 5-hydroxytryptophan and folinic acid treatments had normal neurocognitive outcome. In contrast, the remaining three patients had decreased adherence to a protein restricted diet and medications, and demonstrated mild to severe neurocognitive dysfunction.

### Non-neurotransmitter disorders with a confirmed underlying genetic diagnosis

In this group, a genetic diagnosis was confirmed in 14 patients (9.3%). Patients with normal or non-diagnostic CSF neurotransmitter results underwent targeted single gene testing based on their clinical features. Lumbar puncture guided the diagnosis of glucose transporter 1 (GLUT1) deficiency in one patient with low CSF glucose level and severe methylenetetrahydrofolate reductase (MTHFR) deficiency in another patient with low CSF 5-MTHF level. Three out of 14 patients (21%) had one of the treatable inherited metabolic disorders including cobalamin G, MTHFR and GLUT1 deficiencies. Clinical, biochemical, molecular genetic results of these patients are summarized in Table 4.

**Table 4:** **Patients with non-neurotransmitter disorders with a confirmed inherited metabolic or genetic disease**

**Patients/sex/current age/diagnosis**	**Clinical features/age of onset/age of diagnosis**	CSF* HVA/5-HIAA/3-OMD/othe r (LP age)	**Genetic testing**
1/F/4 yr/MTHFR def.	GDD, epilepsy, tremor/4 d/10 mo	N/**↓112**/N/**↓5-MTHF <5** (1 yr)	Homozygous novel c.379C > T (p.His127Tyr) in *MTHFR* gene
2/F/3 yr/cobolamin G def.	GDD, epilepsy/2 mo/7 mo	**↓245**/**↓109**/N/**↓5-MTHF 28** (4 mo)	Homozygous known [20] c.340_166A > G in *MTR* gene
3/M/11 yr GLUT1 def.	GDD, epilepsy, 2 yr/11 yr	N/N/N/glucose 2.1 (8 yr)	Heterozygous, *de novo,* known [21] c.823G > A (p.Ala275Thr) in *SLC2A1* gene
4/F/16 yr/calcium channelopathy	GDD, epilepsy, ataxia, tremor/15 mo/10 yr	↓123/N/N (10 yr)	Heterozygous, de novo, novel c.2134A > G (p.Ile712Val) in *CACNA1A* gene
5/M/8 yr/calcium channelopathy	GDD, epilepsy, left hemiparesis/6 mo/4 yr	N/N/N (2 yr)	Heterozygous, de novo, novel c.4046G > A (p.Arg1349Glu) in *CACNA1A* gene
6/F/13 yr/ataxia-oculomotor apraxia type 1	GDD, ataxia, dysarthria, epilepsy/ 1.5 yr/12 yr	N/↓50/N (7 yr)	Homozygous 0.132 MB deletion at 9p21.1 in aCGH loss of both copies of *APTX* gene
7/F/19 yr/HSP type 11	GDD, rigidity, spasticity, dystonia/5 yr/18 yr	N/N/N (16 yr)	Heterozygous known [22,23] c.3664_3665insT novel r.4667_4774del (36 AA del) in *SPG11* gene (RT-PCR)
8/M/11 yr/Allen-Herdon-Dudley synd.	GDD, epilepsy, hypotonia/1 yr/9 yr	↓145/N/N (7 yr)	Heterozygous novel c.869C > T (p.Ser290Phe) in *SLC16A2* gene
9/M/passed away/ Prader-Willi synd.	Hypotonia/birth/8 mo following death	N/N/N (2 wk)	Abnormal methylation of *SNRPN* gene
10/F/4 yr/KCNQ2 EE	GDD, epilepsy/1 wk/4.5 yr	N/N/N (1 mo)	Heterozygous, de novo, novel c.700A > C (p.Thr234Pro) in *KCNQ2* gene
11/ F/3 yr/STXBP1 EE	GDD, epilepsy/birth/4 yr	N/N/N (3mo)	Heterozygous, de novo, novel c.364C > T (p.Arg122X) in *STXBP1* gene
12/ M/8 mo/hyperekplexia	GDD, epilepsy, central hypotonia/birth/7 mo	N/N/N (1mo)	Homozygous known [24] c.1274C > T (p.Tyr425Met) in *SLC6A5* gene
13/ M/2 yr/chromosomal abnormality	GDD, epilepsy/birth/1 yr	N/N/N (7mo)	Novel 20q13.33 1.2 Mb deletion on aCGH
14/M/passed away (2 mo)/asparagine synthetase def.	GDD, hypotonia/2 mo	↓240/ ↓183/N (2mo)	Homozygous known [25] c.1648C > T; (p.Arg550Cys) in *ASNS* gene

*Abbreviations*: *CSF* cerebral spinal fluids, *HVA* homovanillic acid, *5-HIAA* 5-hydroxyindol acetic acid, *3-OMD* 3-O-methyldopa, *LP* lumbar puncture, *def*. deficiency, *MTHF* methylenetetrahydrofolate, *d* day(s), *mo* month(s), *yr* year(s), *GDD* global developmental delay, *N* normal, GLUT1 glucose transporter 1. 3: ; ↑ = elevated; ↓ = decreased.

*Age appropriate reference ranges for CSF neurotransmitters, amino acids and glucose: *CSF HVA:* 0–0.2 years = 337–1299 nmol/L; 0.2 - 0.5 years = 450–1132 nmol/L; 0.5 – 2 years = 294 – 1115 nmol/L; 2 – 5 years = 233–928 nmol/L; 5 – 10 years = 218–852 nmol/L; 10 – 15 years = 167–563 nmol/L; adults = 145–324 nmol/L.

*CSF 5HIAA*: 0–0.2 years = 208 – 1159 nmol/L; 0.2 - 0.5 years = 179 – 711 nmol/L; 0.5 - 2 years = 129 – 520 nmol/L; 2 – 5 years = 74 – 345 nmol/L; 5 – 10 years = 66 – 338 nmol/L; 10 – 15 years = 67 – 189; adults = 67–140 nmol/L.

*CSF 3-O-MD*: 0–0.2 years = <300 nmol/L; 0.2 - 0.5 years = <300 nmol/L; 0.5 – 2 years = <300 nmol/L; 2 – 5 years = <150 nmol/L; 5 – 10 years = <100 nmol/L; 10 – 15 years = <100 nmol/L; adults = <100 nmol/L.

*CSF MTHF*: 40–120 nmol/L all age groups.

*CSF glucose:* 2.1-3.6 mmol/L.

In all patients with autosomal recessive disorder, carrier status was confirmed in both parents. In all patients with autosomal dominant disorder, variants occurred *de novo*. None of the novel variants were found in either ESP6500 Exome or dbSNP database as polymorphisms. The variants were highly conserved across all species and reported to be deleterious in SIFT and/or Mutation Taster prediction programs, and all were likely disease causing mutations.

## Discussion

Our retrospective cohort study reports 4% prevalence of inherited neurotransmitter disorders in patients with GDD, neonatal hypotonia, neonatal seizures, epilepsy and movement disorders, who underwent measurements of CSF catecholamine and serotonin metabolites for diagnostic work-up in a single center. Autosomal dominant GTPCH deficiency, TH deficiency, PTPS deficiency, DHPR deficiency, PDE-*ALDH7A1* and PNPO deficiency combining inherited monoamine and pyridoxine metabolism disorders were identified in our study cohort. The prevalence of inherited monoamine metabolism disorders (autosomal dominant GTPCH, TH, DHPR and PTPS deficiencies) was 1.3% (4 out of 150 patients), which is similar compared to a previous study reporting 1.5% prevalence [26]. In a retrospective study of 62 patients who underwent CSF metabolic investigations for neurometabolic disorders, 16 patients (25.8%) were biochemically diagnosed with inherited neurotransmitter disorders [13]. None of these diagnoses were confirmed by molecular genetic investigations and the study included serine biosynthesis defects and cerebral folate deficiency, which are not classified as inherited neurotransmitter disorders [1]. For these reasons, this high prevalence of 25.8% is not a true prevalence study [13]. In our retrospective cohort study, 9.3% (14 out of 150) of the patients with normal or non-diagnostic CSF catecholamine and serotonin metabolite measurements had an identifiable genetic cause other than inherited neurotransmitter disorders (Figure 1). Approximately 5.3% (8 out of 150) of the patients had one of the treatable inherited neurotransmitter disorder or inherited metabolic disorder with favorable short-term neurodevelopmental outcomes, highlighting the importance of an early and specific diagnosis. This is a retrospective cohort study and does not include all patients who underwent lumbar puncture during the study period in our hospital. It might be likely that other neurotransmitter disorders, such as glycine encephalopathy and GABA metabolism disorders, are not captured, which might be diagnosed during this retrospective cohort study period.

The estimated incidence of inherited monoamine metabolism disorders with hyperphenylalaninemia is 1-2% of all patients with a positive screen for PKU in the newborn screening programs [7]. In our province, about 150,000 newborns per year are screened and our hospital is the reference center for about 50% of newborns. In our study, the estimated incidence of PTPS or DHPR deficiencies was about 1:320,000 newborns for each PTPS and DHPR deficiencies. In a recent large international registry of patients with tetrahydrobiopterin metabolism defects (BIODEF; http://www.biopku.org), PTPS deficiency was the most common disorder (56.7%) and DHPR deficiency was the second most common disorder (34.7%) [7]. There are no detailed long-term treatment outcome studies reporting whether DHPR deficiency results in normal neurocognitive outcome. In our small study cohort, there were 5 patients with DHPR deficiency and long-term treatment outcome was available in four patients. One of the patients with strict phenylalanine-restricted diet and l-dopa/carbidopa and 5-hydroxytryptophan treatment had normal neurocognitive outcome at the age of 13 years, whereas, 3 patients with treatment compliance problems had various degrees of neurocognitive dysfunction. Detailed retrospective treatment outcome studies of patients with DHPR deficiency would be necessary to increase our understanding for this treatable disease. Studies are underway to determine, if sapropterin would be helpful to control phenylalanine levels instead of protein restricted diet to decrease further burden to the families as well as to decrease contribution of additional high phenylalanine neurotoxicity.

Autosomal dominant GTPCH deficiency is one of the rare and treatable inherited monoamine metabolism disorders [27]. Patients usually present with dystonia in childhood. Atypical presentations with Parkinsonian features e.g. tremor, have also been reported. In a review article, all patients had low CSF HVA, neopterin, and biopterin levels, and some patients had low to low-normal CSF 5-HIAA levels [27-29]. Our patient presented with tremors at the age of ten years, leading to measurements of CSF neurotransmitter metabolites and the diagnosis of autosomal dominant GTPCH deficiency. Less than 80 patients have been reported since the first description of TH deficiency. According to the recent phenotypic classification [30], our patient had very severe phenotype and was on high dose of L-dopa/carbidopa treatment. The only benefit of this treatment is improvements in choreoathetosis, which is in line with the patients reported in the literature for very severe phenotype of TH deficiency.

Low HVA levels secondary to various neurological disorders have been reported between 6-20% of patients [26,31]. In those studies, between 20-37% of the patients were diagnosed with one of the non-neurotransmitter genetic disorders [26,31]. In our cohort, 8 out of the 150 patients (5.3%) had low HVA and 5 (62.5%) of these patients had an underlying inherited neurotransmitter or non-neurotransmitter disorder with a confirmed underlying genetic diagnosis. In our retrospective cohort study, abnormal CSF neurotransmitter metabolite levels were present in 10 out of 130 (7.7%) patients with no identifiable genetic cause and 6 patients (60%) presented with a movement disorder including ataxia, tremor or dystonia. Interestingly, one of the patients was diagnosed with pyridoxal-phosphate responsive epilepsy based on the low HVA, 5-HIAA and pyridoxal-5-phosphate levels in CSF and responded to pyridoxal-5-phosphate monotherapy with seizure freedom. In this patient PDE-ALDH7A1 and PNPO deficiency have been excluded based on the normal urine alpha-amino adipic semialdehyde, direct sequencing and multiplex ligation dependent probe amplification of the *ALDH7A1* and *PNPO* genes.

Targeted next-generation sequencing for movement disorders utilizing 151-gene panel has been recently reported with a diagnostic yield of 20% compared to targeted single gene Sanger sequencing diagnostic yield of 5% [32]. Targeted next-generation sequencing for epilepsy utilizing 35- 265-gene panels has been previously reported with diagnostic yields ranging between 10–48.5% [33-37]. The clinical targeted next generation epilepsy panels include PDE-ALDH7A1, PNPO and GLUT1 deficiencies and dystonia panels include TH, GTPCH, GLUT1, SR and dopamine transporter deficiencies. Expanding the genes in the targeted next generation dystonia panels including *SLC18A2* (VMAT2 deficiency), *AADC* gene (AADC deficiency), *QDPR (*DHPR deficiency) and *PTS* (PTPS deficiency) would preempt the necessity of CSF neurotransmitter metabolite measurements. Targeted next generation sequencing panels for dystonia and epilepsy would be the most suitable investigation to apply as first line testing in patients with movement disorders and epilepsy rather than using CSF neurotransmitter measurements. In patients with high clinical suspicion of inherited neurotransmitter disorders who have a negative molecular genetic investigation, CSF neurotransmitter measurements could be helpful to guide symptomatic treatment.

## Conclusion

Our retrospective cohort study reports that the prevalence of inherited neurotransmitter disorders is 4% in patients with GDD, neonatal hypotonia, neonatal seizures, epilepsy and movement disorders or newborn screening positive for PKU who underwent CSF neurotransmitter measurements for diagnostic work-up. These disorders include autosomal dominant GTPCH deficiency, TH deficiency, PTPS deficiency, DHPR deficiency, PDE-*ALDH7A1* and PNPO deficiency combining inherited monoamine and pyridoxine metabolism disorders. In our cohort, 5.3% had one of the treatable inherited metabolic disorders with favorable short-term neurodevelopmental outcomes, highlighting the importance of an early and specific diagnosis. Whole exome or genome sequencing might shed light to unravel underlying genetic defects of new inherited neurotransmitter disorders in near future.

## Abbreviations

GABA: Gamma amino butyric acid
CNS: Central nervous system
HVA: Homovanillic acid
5-HIAA: 5-hydroxyindolacetic acid
MHPG: 3-methoxy 4-hydroxypenylglycol
TH: Tyrosine hydroxylase
AADC: Aromatic L-amino acid decarboxylase
GTPCH: Guanosine triphosphate cyclohydrolase I
DHPR: Dihydropteridine reductase
PTPS: 6-pyruvoyltetrahydropterin synthase
SR: Sepiapterin reductase
GDD: Global developmental delay
3-OMD: 3-O-methyldopa
PKU: Phenylketonuria
PDE: Pyridoxine dependent epilepsy
PDE-*ALDH7A1*: Pyridoxine dependent epilepsy in the *ALDH7A1* gene
GLUT1: Glucose transporter 1
MTHFR: Methylenetetrahydrofolate reductase
